# Searching PubMed to Retrieve Publications on the COVID-19 Pandemic: Comparative Analysis of Search Strings

**DOI:** 10.2196/23449

**Published:** 2020-11-26

**Authors:** Jeffrey V Lazarus, Adam Palayew, Lauge Neimann Rasmussen, Tue Helms Andersen, Joey Nicholson, Ole Norgaard

**Affiliations:** 1 Barcelona Institute for Global Health (ISGlobal) Hospital Clínic University of Barcelona Barcelona Spain; 2 Department of Epidemiology, Biostatistics, and Occupational Health McGill University Montreal, QC Canada; 3 Danish Diabetes Knowledge Center Steno Diabetes Center Copenhagen Gentofte Denmark; 4 NYU Langone Health NYU Grossman School of Medicine NYU Health Sciences Library New York, NY United States

**Keywords:** coronavirus, COVID-19, pandemic, scientific publishing, PubMed, literature searching, research, literature, search, performance

## Abstract

**Background:**

Since it was declared a pandemic on March 11, 2020, COVID-19 has dominated headlines around the world and researchers have generated thousands of scientific articles about the disease. The fast speed of publication has challenged researchers and other stakeholders to keep up with the volume of published articles. To search the literature effectively, researchers use databases such as PubMed.

**Objective:**

The aim of this study is to evaluate the performance of different searches for COVID-19 records in PubMed and to assess the complexity of searches required.

**Methods:**

We tested PubMed searches for COVID-19 to identify which search string performed best according to standard metrics (sensitivity, precision, and F-score). We evaluated the performance of 8 different searches in PubMed during the first 10 weeks of the COVID-19 pandemic to investigate how complex a search string is needed. We also tested omitting hyphens and space characters as well as applying quotation marks.

**Results:**

The two most comprehensive search strings combining several free-text and indexed search terms performed best in terms of sensitivity (98.4%/98.7%) and F-score (96.5%/95.7%), but the single-term search COVID-19 performed best in terms of precision (95.3%) and well in terms of sensitivity (94.4%) and F-score (94.8%). The term Wuhan virus performed the worst: 7.7% for sensitivity, 78.1% for precision, and 14.0% for F-score. We found that deleting a hyphen or space character could omit a substantial number of records, especially when searching with SARS-CoV-2 as a single term.

**Conclusions:**

Comprehensive search strings combining free-text and indexed search terms performed better than single-term searches in PubMed, but not by a large margin compared to the single term COVID-19. For everyday searches, certain single-term searches that are entered correctly are probably sufficient, whereas more comprehensive searches should be used for systematic reviews. Still, we suggest additional measures that the US National Library of Medicine could take to support all PubMed users in searching the COVID-19 literature.

## Introduction

Since it was declared a pandemic on March 11, 2020, COVID-19 has dominated headlines around the world and generated thousands of scientific articles [1]. The fast speed of publication has challenged researchers and other stakeholders to keep up with the volume of published articles on the topic [2]. To search the literature, researchers and others use databases of peer-reviewed scientific articles. These databases require indexing and curation of articles, which is a time-consuming task; however, the investment in curated databases aims to establish reliable and more efficient article searching [3].

PubMed is the database of choice for many clinicians and researchers due to its ease of use, reputation, large number of indexed journals, and free access [4]. It is maintained by the US National Library of Medicine (NLM) [4-6]. PubMed provides subject filters to facilitate searching specific topics but as they are only reviewed once a year, it is unknown if a COVID-19 subject filter is under consideration [7]. However, at the beginning of the pandemic, NLM introduced a one-click search option on their dedicated website for COVID-19/SARS-CoV-2 [8]. By clicking on a button, PubMed users can quickly apply a ready-made search to identify potentially relevant COVID-19 records in PubMed. Searching structured databases like PubMed is generally considered to be most skillfully executed by employing both Medical Subject Headings (MeSH) and free-text terms [9-11]. However, conducting systematic, comprehensive, and transparent searches takes time and skill [12-14]. Thus, PubMed’s one-click search is potentially a valuable shortcut for users worldwide dealing with the COVID-19 pandemic.

A recent survey showed that authors of systematic reviews have different perspectives on what constitutes an “effective” literature search [15]. Based on the survey, Cooper et al [15] note that review authors “appeared to locate effectiveness within a binary classification between types of review which are either comprehensive or non-comprehensive.” In short, their article indicates that those valuing comprehensive literature searches tend to be literature searchers (eg, information specialists or librarians) who emphasize sensitivity as an evaluation metric for effectiveness and highlight Cochrane-style systematic reviews, with their ambition to identify all available evidence as an ideal. On the other hand, researchers and health professionals screening the literature tend to emphasize less comprehensive searching and value precision as an evaluation metric. In addition, they care about outcomes like the workload, time, and resources that are needed to handle identified records. However, most PubMed users only browse the first 20 records of their search results and refine their searches to make the number of hits manageable [3]. For such reasons, the NLM also has an algorithm that is designed to sort search results according to their relevance via the Best Match sorting option [16] and offers one-click filters (eg, publication date and article type). These user behaviors and options reflect a different view on literature searches than those voiced by users conducting formal literature reviews [15]. This might be because PubMed users also consist of clinicians, health professionals, and other decision makers that search the literature not to conduct reviews but with other types of evidence use in mind.

No matter one’s perspective, the effectiveness of any literature search depends on the semantic variability related to the question that the search is to inform. When a research topic is in flux, establishing common terminology is crucial to identify relevant records. A retrospective study of searching PubMed during the first 10 weeks of the 2009 H1N1 influenza pandemic showed that inconsistent disease naming and a lag in indexing increased the risk of missing relevant studies when searching the scientific literature during the 2009 pandemic [17]. The authors of the study recommended that, at the start of a pandemic, “the international scientific community should agree on nomenclature and the specific name to be used earlier, and the U.S. National Library of Medicine and other database providers should incorporate that in their indexing of all relevant articles” [17]. Regarding the COVID-19 pandemic, the naming of the disease occurred relatively early. On February 11, 2020, the International Committee on Taxonomy of Viruses officially named the novel coronavirus SARS-CoV-2 and the World Health Organization (WHO) named the disease COVID-19 [18]. However, article authors still use several other terms for the virus and the disease, such as simply “coronavirus” and, earlier, “Wuhan coronavirus” or “Wuhan virus.”

In this study, we aimed to evaluate the performance of different searches for COVID-19 records in PubMed and to assess the complexity of searches that was required. Therefore, we compared the performance of PubMed’s one-click search option with both simpler and more complex search strings for the first 10 weeks of the COVID-19 pandemic. We also examined the deletion of hyphens or spaces as well as quotation marks from the simple searches to mimic potential user errors or preferences, such as variant spellings of words and the tendency to use only simple keyword searches [19]. Finally, we discuss the searches in relation to the varying perspectives on search effectiveness outlined above to make recommendations on how NLM can improve PubMed.

## Methods

### General Methodology

We constructed a comprehensive COVID-19 search string and compared it to seven other related search strings (Table 1). We queried PubMed for each of these different searches and calculated their sensitivity, precision, and F-score using a COVID-19 database (LitCovid) maintained and curated by an NLM branch as a gold standard [20]. We then used these calculated values to compare the performance of the different search strings.

**Table 1:** Search strings and translations of the different searches.

Search title	Entered search	Translation in PubMed
Our comprehensive search (Search 1)	(“COVID-19”[nm] OR “COVID-19 diagnostic testing”[nm] OR “COVID-19 drug treatment”[nm] OR “COVID-19 serotherapy”[nm] OR “COVID-19 vaccine”[nm] OR “severe acute respiratory syndrome coronavirus 2”[nm] OR 2019-ncov*[tiab] OR 2019ncov*[tiab] OR 2019-novel-cov*[tiab] OR coronavirus[ti] OR coronavirus-2*[tiab] OR coronavirus-disease-19*[tiab] OR corona-virus-disease-19*[tiab] OR coronavirus-disease-20*[tiab] OR corona-virus-disease-20*[tiab] OR covid-19*[tiab] OR covid19*[tiab] OR covid-20*[tiab] OR covid20*[tiab] OR ncov-2019*[tiab] OR ncov2019*[tiab] OR new-coronavirus[tiab] OR new-corona-virus[tiab] OR novel-coronavirus[tiab] OR novel-corona-virus[tiab] OR sars-2*[tiab] OR sars2*[tiab] OR sars-cov-19*[tiab] OR sars-cov19*[tiab] OR sarscov19*[tiab] OR sarscov-19*[tiab] OR sars-cov-2*[tiab] OR sars-cov2*[tiab] OR sarscov2*[tiab] OR sarscov-2*[tiab] OR ((“Coronavirus”[mh] OR “Coronavirus Infections”[mh] OR betacoronavirus[tiab] OR beta-coronavirus[tiab] OR beta-corona-virus[tiab] OR corona-virus[tiab] OR coronavirus[tiab] OR sars*[tiab] OR severe-acute-respiratory*[tiab]) AND (2019[tiab] OR 2020[tiab] OR wuhan*[tiab] OR hubei*[tiab] OR china*[tiab] OR chinese*[tiab] OR outbreak*[tiab] OR epidemic*[tiab] OR pandemic*[tiab]))) AND 2019/12:3000[dp]	None
Shokraneh’s [21] comprehensive search (Search 2)	((((((((((((((((((((((“Betacoronavirus”[MeSH Terms] OR “Coronavirus Infections”[MeSH Terms]) OR “COVID-19”[Supplementary Concept]) OR “Coronavirus”[MeSH Terms]) OR “Severe Acute Respiratory Syndrome Coronavirus 2”[Supplementary Concept]) OR “2019nCoV”[All Fields]) OR “betacoronavirus*”[All Fields]) OR “corona virus*”[All Fields]) OR “coronavirus*”[All Fields]) OR “coronovirus*”[All Fields]) OR “CoV”[All Fields]) OR “CoV2”[All Fields]) OR “COVID”[All Fields]) OR ((“COVID-19”[Supplementary Concept] OR “COVID-19”[All Fields]) OR “covid19”[All Fields])) OR (((((((“COVID-19”[All Fields] OR “covid 2019”[All Fields]) OR “Severe Acute Respiratory Syndrome Coronavirus 2”[Supplementary Concept]) OR “Severe Acute Respiratory Syndrome Coronavirus 2”[All Fields]) OR “2019 ncov”[All Fields]) OR “SARS CoV 2”[All Fields]) OR “2019nCoV”[All Fields]) OR ((“wuhan”[All Fields] AND (“Coronavirus”[MeSH Terms] OR “Coronavirus”[All Fields])) AND (2019/12/1:2019/12/31[Date - Publication] OR 2020/1/1:2020/12/31[Date - Publication])))) OR “HCoV-19”[All Fields]) OR “nCoV”[All Fields]) OR “SARS CoV 2”[All Fields]) OR “SARS2”[All Fields]) OR “SARSCoV”[All Fields]) OR ((((“sars virus”[MeSH Terms] OR (“sars”[All Fields] AND “virus”[All Fields])) OR “sars virus”[All Fields]) OR (“sars”[All Fields] AND “CoV”[All Fields])) OR “sars cov”[All Fields])) OR ((“Severe Acute Respiratory Syndrome Coronavirus 2”[Supplementary Concept] OR “Severe Acute Respiratory Syndrome Coronavirus 2”[All Fields]) OR “SARS CoV 2”[All Fields])) OR “severe acute respiratory syndrome cov*”[All Fields]) AND (2019/11/17:3000/12/31[Date - Entry] OR 2019/11/17:3000/12/31[Date - Publication])	None
One-click search (Search 3)	((wuhan[All Fields] AND (“coronavirus”[MeSH Terms] OR “coronavirus”[All Fields])) AND 2019/12[PDAT] : 2030[PDAT]) OR 2019-nCoV[All Fields] OR 2019nCoV[All Fields] OR COVID-19[All Fields] OR SARS-CoV-2[All Fields]	(((((“wuhan”[All Fields] AND (“coronavirus”[MeSH Terms] OR “coronavirus”[All Fields])) AND 2019/12/1:2030/12/31[Date - Publication]) OR ((“severe acute respiratory syndrome coronavirus 2”[Supplementary Concept] OR “severe acute respiratory syndrome coronavirus 2”[All Fields]) OR “2019 ncov”[All Fields])) OR “2019nCoV”[All Fields]) OR (((((((“covid 19”[All Fields] OR “covid 2019”[All Fields]) OR “severe acute respiratory syndrome coronavirus 2”[Supplementary Concept]) OR “severe acute respiratory syndrome coronavirus 2”[All Fields]) OR “2019 ncov”[All Fields]) OR “sars cov 2”[All Fields]) OR “2019nCoV”[All Fields]) OR ((“wuhan”[All Fields] AND (“coronavirus”[MeSH Terms] OR “coronavirus”[All Fields])) AND (2019/12/1:2019/12/31[Date - Publication] OR 2020/1/1:2020/12/31[Date - Publication])))) OR ((“severe acute respiratory syndrome coronavirus 2”[Supplementary Concept] OR “severe acute respiratory syndrome coronavirus 2”[All Fields]) OR “sars cov 2”[All Fields])
Single-term search for COVID-19 (Search 4)	COVID-19	“COVID-19”[All Fields] OR “COVID-2019”[All Fields] OR “severe acute respiratory syndrome coronavirus 2”[Supplementary Concept] OR “severe acute respiratory syndrome coronavirus 2”[All Fields] OR “2019-nCoV”[All Fields] OR “SARS-CoV-2”[All Fields] OR “2019nCoV”[All Fields] OR ((“Wuhan”[All Fields] AND (“coronavirus”[MeSH Terms] OR “coronavirus”[All Fields])) AND (2019/12[PDAT] OR 2020[PDAT]))
Single-term search for SARS-CoV-2 (Search 5)	SARS-CoV-2	“severe acute respiratory syndrome coronavirus 2”[Supplementary Concept] OR “severe acute respiratory syndrome coronavirus 2”[All Fields] OR “sars cov 2”[All Fields]
Single-term search for Coronavirus (Search 6)	Coronavirus	“coronavirus”[MeSH Terms] OR “coronavirus”[All Fields] OR “coronaviruses”[All Fields]
Single-term search for Wuhan coronavirus (Search 7)	Wuhan coronavirus	“severe acute respiratory syndrome coronavirus 2”[Supplementary Concept] OR “severe acute respiratory syndrome coronavirus 2”[All Fields] OR “wuhan coronavirus”[All Fields]
Single-term search for Wuhan virus (Search 8)	Wuhan virus	(“Wuhan”[All Fields] AND ((((((“virology”[MeSH Subheading] OR “virology”[All Fields]) OR “viruses”[All Fields]) OR “viruses”[MeSH Terms]) OR “virus s”[All Fields]) OR “viruse”[All Fields]) OR “virus”[All Fields]))

### Constructing a Comprehensive Search String

A comprehensive search string (Search 1) was initially developed by ON, LNR, and THA to monitor newly published COVID-19–related studies as part of their work at the Danish Diabetes Knowledge Center. Subsequently, the search string was revised based on the authors’ subject knowledge, analyses of free-text words and MeSH terms conducted in PubReMiner [22], and search strings developed by information specialists that were made publicly available (eg, by the Medical Library Association and different university libraries). Several versions were tested and reiterated before the final version was reached [14]. The final search string was then reviewed by AP and JVL.

### Comparing Search Strings

In addition to Search 1, we conducted Searches 2 through 8, which included an additional comprehensive search developed by Shokraneh (Search 2) [21], the one-click search option developed by NLM (Search 3), as well as five common terms used to search PubMed for COVID-19–related records (Searches 4-8), to compare different comprehensive searches and compare the comprehensive searches to the simple PubMed queries [21] (Table 1). The automatic term-mapping feature in PubMed translates some of these basic queries to more comprehensive search strings that include synonyms and MeSH terms, as shown in Table 1. We observed that the COVID-19 translation does not seem to follow the standard mapping process for automatic term mappings [23].

### Simulating Historical Weekly Searches

We searched from March 11 to May 19, 2020, spanning a total of 10 weeks, or 70 days, from when the WHO declared COVID-19 a pandemic. We limited the searches to find only records registered in PubMed during each of the 10 weeks (Wednesday through Tuesday for each week). For each of the eight searches, we recorded and analyzed the weekly number of records by using the date of the records’ entry to the PubMed database (EDAT field in PubMed). All searches were conducted in the current standard version of PubMed on June 26, 2020.

### Analysis of the Searches

We compared the evaluation metrics sensitivity, precision, and F-score for each search. Sensitivity is a measure of search effectiveness and is defined as the number of relevant records in the gold standard data set identified by the search (true positives) over all relevant records in the gold standard data set (true positives and false negatives) [24]. Sensitivity is also known as recall; however, to describe literature search effectiveness, the term sensitivity is widely used (eg, by NLM when reporting the effectiveness of PubMed Clinical Queries Filters) [24,25]. Precision is a measure of search efficiency and is defined as the number of relevant records identified by the search (true positives) over the total number of identified records (true positives and false positives). Finally, the F-score is defined as the harmonic mean of the sensitivity and the precision (Table 2). We used the LitCovid database as the gold standard to compare the PubMed searches against [20]. This database contains COVID-19–related records solely from PubMed and is curated by the NLM based on daily broad PubMed searches. As we do not know which records were deemed not relevant by the NLM, we do not know the number of true negatives. Thus, it was not possible to reliably calculate other relevant metrics, such as the specificity and accuracy of the evaluated search strings. For the calculations of the evaluation metrics, the searches were rerun, limiting the dates from January 17, 2020 (the earliest date of any record in LitCovid), to May 19, 2020 (the end of the study period). For this period, the LitCovid database contains 14,018 records.

**Table 2:** Descriptions and calculations of metrics.

Metric	Description	Calculation^a^
Sensitivity	The probability that the search identified a record as relevant given that the record is relevant; also known as recall.	T^+^D^+^/(T^+^D^+^+T^–^D^+^)
Precision	The probability that the record is relevant given that the record was identified as relevant; also known as positive predictive value (PPV). The number needed to read (NNR) can also be calculated by 1/precision.	T^+^D^+^/(T^+^D^+^+T^+^D^–^)
F-score	The harmonic mean of the sensitivity and precision.	2 × sensitivity × precision/(sensitivity + precision)

^a^We denote T^+^ as in the search string as compared to the LitCovid database, T^–^ as not in the search string as compared to the LitCovid database, D^+^ as present in the LitCovid database as compared to the search string, and D^–^ as not present in the LitCovid database as compared to the search string.

### Sensitivity to the Deletion or Addition of Hyphens, Spaces, and Quotation Marks

Spelling mistakes have previously been documented to affect PubMed results [26]. Further, users of PubMed may have different writing style preferences and choose to apply or leave out hyphens and spaces. To investigate the possible implications of entering different versions of the search terms COVID-19 (Search 4) and SARS-CoV-2 (Search 5), we compared the results after omitting a hyphen and/or a space (eg, COVID19, COVID 19, COVID-19). We also assessed the implications of surrounding search terms with quotation marks, assuming that some users may do this to run a highly specific search (eg, “COVID-19,” “COVID19,” “COVID 19”). The number of identified records were documented for each version of the term.

### Proportion of MEDLINE-Indexed Records

To investigate the potential use of the indexing that is done when records are indexed in MEDLINE (ie, adding indexing terms such as MeSH and Supplementary Concepts), we calculated the proportion of records that had been MEDLINE-indexed out of the total number of records present in the LitCovid database by May 19, 2020. This was done by querying PubMed twice to retrieve the first 9999 PubMed IDs in the LitCovid database, followed by a second query to retrieve the remaining articles. We then looked at how many of the retrieved records out of the total number of retrieved records were tagged with the STAT – MEDLINE tag, indicating the status of the record as indexed in the MEDLINE database.

### Software Analysis and Reproducibility Statement

All analyses were run in R (Version 4.0.2; R Foundation for Statistical Computing) and data were stored in text files when downloaded from PubMed using the PubMed format option. All search strings, code, and data to reproduce this analysis are available [27].

## Results

### Overview

**Figure 1:**
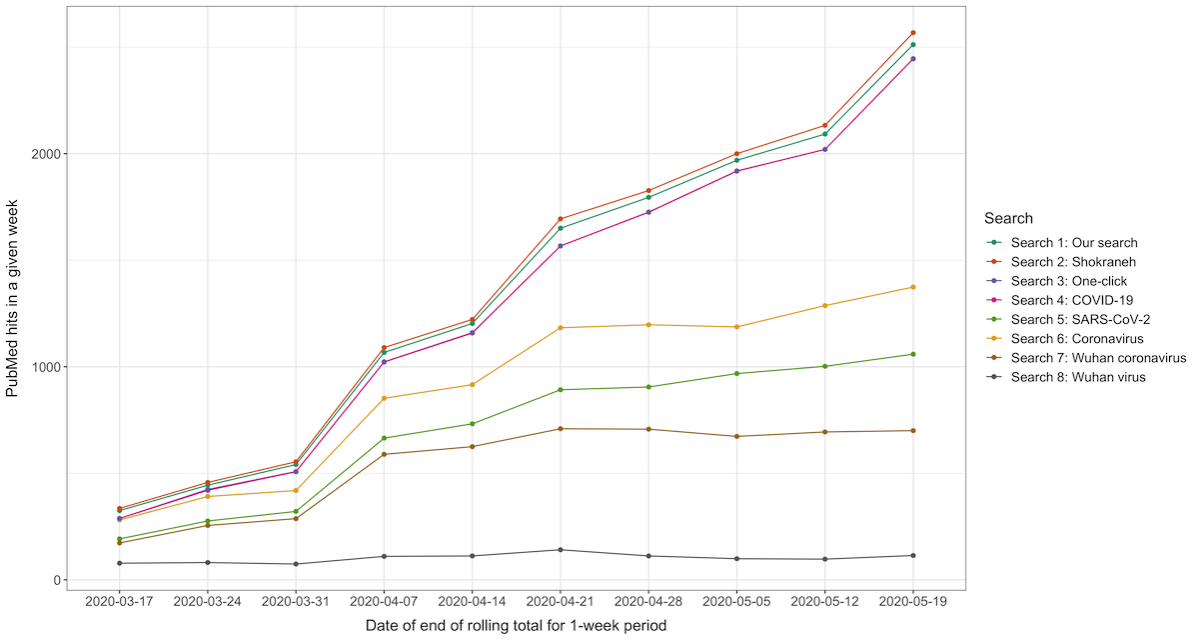
Records found over time from March 11 to May 19, 2020. Search 3, the one-click search, is not visible on the graph as it matches the results of the single-term search using COVID-19 (Search 4).

In total, over the 10-week period studied, we found 13,599 records with our comprehensive search (Search 1). The Shokraneh search (Search 2; see Methods) found the largest number of records (n=13,880). The one-click search (Search 3) and the single-term search for COVID-19 (Search 4) yielded the same results, with the third-highest number of records (n=13,071). Next, the single-term search for Coronavirus (Search 6) found 9087 records, which was the fifth-highest number, and the single-term search for SARS-CoV-2 (Search 5) found 7012 records, which was the sixth-highest number. The Wuhan coronavirus search (Search 7) found 5412 records and the Wuhan virus search (Search 8) found 1013 records. The number of records per week for each of the search strings is shown in Figure 1.

### Evaluation of Search Strings

We found that the comprehensive searches (Searches 1 and 2) had the highest sensitivities and F-scores compared to all other searches. The searches for SARS-CoV-2 (Search 5) and Wuhan coronavirus (Search 7) had the highest precision (Table 3). The Wuhan virus search (Search 8) had the lowest sensitivity and precision, but otherwise the precision was similar for all the other searches. The difference in sensitivity of 4.0% between our comprehensive search (Search 1) and the single-term search for COVID-19 (Search 4) would translate to an average of 40 excess relevant records missed per 1000 articles identified when comparing the two searches over multiple theoretical sets of 1000 relevant COVID-19 records. This equates to an average of 43 excess relevant records missed if the single-term search COVID-19 (Search 4) was compared against Shokraneh’s comprehensive search (Search 2) over multiple theoretical sets of 1000 relevant COVID-19 records.

**Table 3:** Metrics for the different strings as compared against the LitCovid gold standard.

	Records (n)	Sensitivity (%)	Precision (%)	F-score (%)
Search 1: Our comprehensive search	13,599	98.4	94.6	96.5
Search 2: Shokraneh’s comprehensive search	13,880	98.7	92.7	95.7
Search 3: One-click search	13,071	94.4	95.3	94.8
Search 4: COVID-19	13,071	94.4	95.3	94.8
Search 5: SARS-CoV-2	7012	52.0	96.4	67.6
Search 6: Coronavirus	9087	67.2	93.4	78.3
Search 7: Wuhan coronavirus	5412	40.8	96.4	57.3
Search 8: Wuhan virus	1013	7.7	78.1	14.0

### Sensitivity to Deleting Spaces and Hyphens and Adding Quotation Marks

We observed that automatic term mapping was sensitive to the deletion of hyphens and spaces, especially variations of SARS-CoV-2 (Table 4). We also found a decrease in records if a space or hyphen was removed from the search terms, such as COVID-19 versus COVID19 (13,071 versus 12,607). Furthermore, when quotation marks were added (eg, “COVID19”), the number of results fell to 11,716 records. Once again, SARS-CoV-2 terms were more sensitive to variations than COVID-19 terms.

The proportion of MEDLINE-indexed records out of the total number of records added to the LitCovid database during the studied time period was 49.0% when verified in PubMed on July 5, 2020, for the PMIDs in the LitCovid data set from May 19, 2020.

**Table 4:** Analysis of searches with and without hyphens, spaces, and quotation marks.

Term	Result	Translation
covid-19	13,071	(“COVID-19”[All Fields] OR “COVID-2019”[All Fields] OR “severe acute respiratory syndrome coronavirus 2”[Supplementary Concept] OR “severe acute respiratory syndrome coronavirus 2”[All Fields] OR “2019-nCoV”[All Fields] OR “SARS-CoV-2”[All Fields] OR “2019nCoV”[All Fields] OR ((“Wuhan”[All Fields] AND (“coronavirus”[MeSH Terms] OR “coronavirus”[All Fields]))
covid 19	13,071	(“COVID-19”[All Fields] OR “COVID-2019”[All Fields] OR “severe acute respiratory syndrome coronavirus 2”[Supplementary Concept] OR “severe acute respiratory syndrome coronavirus 2”[All Fields] OR “2019-nCoV”[All Fields] OR “SARS-CoV-2”[All Fields] OR “2019nCoV”[All Fields] OR ((“Wuhan”[All Fields] AND (“coronavirus”[MeSH Terms] OR “coronavirus”[All Fields]))
covid19	12,607	“COVID-19”[Supplementary Concept] OR “COVID-19”[All Fields] OR “covid19”[All Fields]
“covid-19”	12,548	“covid-19”[All Fields]
“covid 19”	12,548	“covid 19”[All Fields]
“covid19”	11,716	“covid19”[All Fields]
sars-cov-2	7055	“severe acute respiratory syndrome coronavirus 2”[Supplementary Concept] OR “severe acute respiratory syndrome coronavirus 2”[All Fields] OR “sars cov 2”[All Fields]
sars cov 2	7055	“severe acute respiratory syndrome coronavirus 2”[Supplementary Concept] OR “severe acute respiratory syndrome coronavirus 2”[All Fields] OR “sars cov 2”[All Fields]
sars-cov 2	7055	“severe acute respiratory syndrome coronavirus 2”[Supplementary Concept] OR “severe acute respiratory syndrome coronavirus 2”[All Fields] OR “sars cov 2”[All Fields]
sars cov-2	7055	“severe acute respiratory syndrome coronavirus 2”[Supplementary Concept] OR “severe acute respiratory syndrome coronavirus 2”[All Fields] OR “sars cov 2”[All Fields]
sarscov-2	17	sarscov-2[All Fields]
sarscov 2	3008	sarscov[All Fields] AND 2[All Fields]
sars-cov2	153	sars-cov2[All Fields]
sars cov2	219	sars[All Fields] AND cov2[All Fields]
sarscov2	3601	sarscov2[All Fields]
“sars-cov-2”	3587	“sars-cov-2”[All Fields]
“sars cov 2”	3587	“sars cov 2”[All Fields]
“sars-cov 2”	3587	“sars-cov 2”[All Fields]
“sars cov-2”	3587	“sars-cov-2”[All Fields]
“sarscov-2”	17	“sarscov-2”[All Fields]
“sarscov 2”	17	“sarscov 2”[All Fields]
“sars-cov2”	153	“sars-cov2”[All Fields]
“sars cov2”	153	“sars cov2”[All Fields]
“sarscov2”	3601	“sarscov2”[All Fields]

## Discussion

In this study, we evaluated eight PubMed searches and examined the differences between 24 alternative single-term searches with and without hyphens, spaces, and quotation marks. We found that the comprehensive search strings performed best in terms of sensitivity and F-score, while the one-click and single-term COVID-19 searches performed almost as well as the comprehensive search in terms of sensitivity and as well as the comprehensive search in terms of precision. The performance of the single-term COVID-19 search is dependent on PubMed’s term mapping that translates the single-term search into a more comprehensive search. Comparatively, searching with SARS-CoV-2 as a single term while relying on the automatic term-mapping feature of PubMed yielded worse results than when searching with the single term COVID-19.

Using the LitCovid database, which covers both COVID-19 (the disease) and SARS-CoV-2 (the virus), as the gold standard comparator might have skewed our results in favor of the COVID-19 automatic term mapping. This is because the mapping translates the single-term search for COVID-19 to terms related to both the disease and the virus, whereas the single-term search for SARS-CoV-2 is translated to terms related only to the virus and not the disease. This dual mapping does have precedent; PubMed’s term mapping often maps the disease terms both to the virus and the disease, whereas the virus terms typically map only to the virus and not the disease.

The relatively early naming of the novel coronavirus, especially the name “COVID-19,” set by the WHO, appears to have facilitated both the widespread use of the COVID-19 term in publications, as we found, and well-performing automatic term mapping in PubMed. As such, the results presented in this study highlight important progress in PubMed searching since the 2009 H1N1 influenza pandemic [17]. This progress could be further improved by NLM extending the mapping of the term COVID-19 to terms elucidated by evaluation of the two comprehensive searches in our analyses.

The analyses are based on our choices of the simple search terms we hypothesized users might enter. PubMed users might use other terms, such as Wuhan Pneumonia or COVID-2019 [28]. Nonetheless, the terms chosen by us consistently illustrate the differences between comprehensive and less comprehensive searches, whether constructed by users or via PubMed’s automatic term mapping. Another limitation is the fact that MEDLINE indexing happens at different points in time after the record has been added to the PubMed database. This may result in different search results depending on the date of the search. Dates for all search results used in this study have been reported where appropriate. Finally, we have observed that the one-click search option has been changed since we conducted our analysis. Currently, the one-click search and the COVID-19 single-term search are identical. Thus, this has no implications on the interpretation of our result and conclusion.

Our sensitivity analyses of hyphens, spaces, and quotation marks also indicate room for improvement, especially when using SARS-CoV-2 as a single term for searching. As different writing style preferences and mistakes are unavoidable (eg, use of hyphens and spaces), automatic term mapping would be improved by being sensitive to this, just as it is sensitive to British and American spelling [23]. Surrounding the search term(s) with quotation marks forces an all-fields term or phrase search for the exact term(s) entered and does not activate automatic term mapping. This can markedly reduce the number of potentially relevant records in the search result as compared with a similar search based on a search string generated by automatic term mapping. The ability to turn off the automatic term mapping by adding quotation marks is not something that needs to be changed as it is a feature in PubMed. However, PubMed users need to be aware that adding quotation marks lowers sensitivity. In addition, the sensitivity analyses should motivate NLM to consider whether all variations (eg, COVID19) should activate the same automatic term mapping as COVID-19 (Search 4).

### Implications for PubMed Users and NLM

Although some emphasize the importance of highly sensitive search strings more than others [15], it would be misleading to argue that the ability to identify all relevant articles on a given topic is relevant only for those conducting comprehensive, systematic reviews. Reviewers who conduct rapid reviews could save time and resources without substantially compromising sensitivity and precision by using the search string from the one click-option. Everyday users of PubMed will need to specify the one click-search to reach the number of records they find manageable and relevant for their situation. However, the validity of the Best Match sorting option in PubMed rests on the sensitivity of the search process. Thus, the benefits of identifying all relevant records can extend to noncomprehensive PubMed searches.

Still, those who aim to conduct Cochrane-style systematic reviews would want to develop more comprehensive search strings rather than relying on the string integrated in the one click-option. Here, PubMed’s Supplementary Concepts implemented for COVID-19 and SARS-CoV-2 could, if correct and consistently applied to all relevant records, help literature searchers conduct efficient searches. However, Supplementary Concepts are applied only to records available in PubMed that have been indexed in MEDLINE, which account for 49.0% of the total records identified in LitCovid. As reported above, the one-click PubMed search yielded the same results as the COVID-19 single-term search, although the latter included the Supplementary Concepts for both COVID-19 and SARS-CoV-2, suggesting that they do not add value when a search string is sufficiently comprehensive. For Supplementary Concepts to be of value for PubMed users aiming to conduct comprehensive reviews during the pandemic, NLM would have to speed up indexing of all records relevant to COVID-19.

We recommend that NLM uses a highly sensitive comprehensive search string to create a COVID-19 subject filter (ie, covid-19[sb]) or add it to their special queries collection [29,30]. The search string incorporated into such a filter or special query may even be activated by the automatic term mapping of a single-term search for COVID-19. If using one of the comprehensive search strings tested in this article to create the subject filter, it should be tested against other gold standard data sets for validation [31]. Future research should test more comprehensive search strings to determine which one is best suited for searching the literature base.

Further, we highlight the need for evaluating and validating search strings on multiple subjects (not only COVID-19) to develop more subject filters, which can be helpful for both everyday informational needs and serve as inspiration when conducting systematic reviews. Even so, we recommend that users consult with information specialists, research librarians, or researchers with the proper competencies for the retrieval of scientific information.

### Conclusions

Scientific evidence must be easily accessible, especially during a pandemic. Overall, we found that changes have been made in PubMed that improve access to COVID-19–related articles compared to the situation during the 2009 H1N1 influenza pandemic. Importantly, some single-term searches performed well. Still, more can be done to support users searching for evidence regarding COVID-19. Specifically, the term mapping of the single-term COVID-19 search can be refined to be sensitive to variations in hyphens and spaces, and highly sensitive comprehensive search strings could be made more easily available for instant application when using the PubMed search interface.

Overall, PubMed users can reliably use the one-click or single-term COVID-19 search for everyday informational needs about COVID-19 and SARS-CoV-2. However, when users are aiming to systematically locate and screen the total available literature on a topic related to COVID-19, especially when conducting systematic reviews, they should rely on comprehensive searches.

## Abbreviations

MeSH: Medical Subject Headings
NLM: National Library of Medicine
WHO: World Health Organization
